# Cost and affordability of non-communicable disease screening, diagnosis and treatment in Kenya: Patient payments in the private and public sectors

**DOI:** 10.1371/journal.pone.0190113

**Published:** 2018-01-05

**Authors:** Sujha Subramanian, Robai Gakunga, Joseph Kibachio, Gladwell Gathecha, Patrick Edwards, Elijah Ogola, Gerald Yonga, Naftali Busakhala, Esther Munyoro, Jeremiah Chakaya, Nancy Ngugi, Nyawira Mwangi, Daniel Von Rege, Lili-Marie Wangari, David Wata, Robert Makori, Julius Mwangi, Walter Mwanda, on behalf of the East African Economics and Implementation Group (EAEIG)

**Affiliations:** 1 Public Health Research Division, RTI International, Waltham, MA, United States of America; 2 Independent Research Scientist, Nairobi, Kenya; 3 Non-Communicable Disease Department, Ministry of Health, Nairobi, Kenya; 4 Department of Clinical Medicine, University of Nairobi, Nairobi, Kenya; 5 Department of Cardiology, Aga Khan University, Nairobi, Kenya; 6 Division of Hematology and Oncology, Moi University, Eldoret, Kenya; 7 Kenyatta National Hospital, Nairobi, Kenya; 8 Kenyatta University, Nairobi, Kenya; 9 Kenya Medical Training College/London School of Hygiene and Tropical Medicine, Nairobi, Kenya; 10 Médecins Sans Frontières, Belgium, Nairobi, Kenya; 11 Department of Human Pathology, University of Nairobi, Nairobi, Kenya; Boston University School of Public Health, UNITED STATES

## Abstract

**Introduction:**

The prevalence of non-communicable diseases (NCDs) is rising in low- and middle-income countries, including Kenya, disproportionately to the rest of the world. Our objective was to quantify patient payments to obtain NCD screening, diagnosis, and treatment services in the public and private sector in Kenya and evaluate patients’ ability to pay for the services.

**Methods and findings:**

We collected payment data on cardiovascular diseases, diabetes, breast and cervical cancer, and respiratory diseases from Kenyatta National Hospital, the main tertiary public hospital, and the Kibera South Health Center—a public outpatient facility, and private sector practitioners and hospitals. We developed detailed treatment frameworks for each NCD and used an itemization cost approach to estimate payments. Patient affordability metrics were derived from Kenyan government surveys and national datasets.

Results compare public and private costs in U.S. dollars. NCD screening costs ranged from $4 to $36, while diagnostic procedures, particularly for breast and cervical cancer, were substantially more expensive. Annual hypertension medication costs ranged from $26 to $234 and $418 to $987 in public and private facilities, respectively. Stroke admissions ($1,874 versus $16,711) and dialysis for chronic kidney disease ($5,338 versus $11,024) were among the most expensive treatments. Cervical and breast cancer treatment cost for stage III (curative approach) was about $1,500 in public facilities and more than $7,500 in the private facilities. A large proportion of Kenyans aged 15 to 49 years do not have health insurance, which makes NCD services unaffordable for most people given the overall high cost of services relative to income (average household expenditure per adult is $413 per annum).

**Conclusions:**

There is substantial variation in patient costs between the public and private sectors. Most NCD diagnosis and treatment costs, even in the public sector, represent a substantial economic burden that can result in catastrophic expenditures.

## Introduction

The prevalence of non-communicable diseases (NCDs) is rising in low- and middle-income countries (LMICs) disproportionate to the rest of the world. Currently, NCDs cause over 36 million annual deaths globally; 14 million of these are premature mortality (among those younger than 70 years), and 90% of these premature deaths occur in LMICs [1]. Cancer, cardiovascular disease, respiratory disease, and diabetes are the major causes of NCD deaths in LMICs [2].

In Kenya, the mortality and morbidity from NCDs is rapidly increasing [3]. Cardiovascular diseases are the leading cause of NCD mortality in Kenya because of the high prevalence of multiple risk factors, including hypertension, diabetes, cholesterol, smoking, and obesity [4]. Multiple studies performed in selected populations across Kenya have identified a high prevalence of hypertension [4–6]. In the first nationally representative survey, performed in 2015 and which included hypertension measurement [7], hypertension was identified in 23.8% of the respondents aged 18 to 69 years and 7% of those not on medication were diagnosed with severe hypertension. Among those aged 18 to 44 years, 10.4% had three or more risk factors for cardiovascular disease, and among those aged 45 to 69 years, 25.9% had three or more risk factors.

Cancer is the second leading cause of NCD mortality, and the incidence of cancer increased from 28,000 to 41,000 between 2008 and 2012 [8, 9]. The three leading cancer sites are the cervix (40.1 cases per 100,000 individuals), the breast (38.3 cases per 100,000 individuals), and the prostate (31.6 cases per 100,000 individuals) [9]. It is observed that most cancer cases are diagnosed at an advanced stage when curative treatment options are limited [10]. There is sparse prevalence data on respiratory diseases—asthma and chronic obstructive pulmonary disease (COPD). A systematic analysis estimated the prevalence of asthma in Africa as 12.8% (95% confidence interval 8.2–17.1) in 2010 [11].

In response to the escalating burden of NCDs in Kenya, the government has established an NCD division within the Ministry of Health. Kenya also launched a 5-year National NCD Strategy in 2015 to guide the implementation of interventions to reduce the mortality from NCDs. To operationalize this strategy, it is important to understand the cost of NCD health services along the continuum of care, including those related to screening, diagnosis, and treatment. Knowledge on the costs of NCD management is needed to identify cost-effective solutions and prioritize interventions to address NCDs. Additionally, it is critically important to assess affordability of NCD services to evaluate patient access to required care. Individuals are required to pay for health care services in the public sector in Kenya; the payments are subsidized, but nevertheless, all services require some level of patient payments. Approximately half of all the health facilities in Kenya are managed by either private for-profit or not-for-profit organizations; therefore, a substantial proportion of health care is obtained in the private sector [12]. A minority if individuals in Kenya have health insurance coverage and almost all employees in the formal sector, which is less than one-fifth of those employed, are covered through the National Hospital Insurance Fund (NHIF). The NHIF provides payments for specific inpatient and outpatient services but not all costs in the private sector are covered and therefore patients still incur out-of-pocket payments for health services. [13]

The objective of this study was to quantify patient payments for NCD screening, diagnosis, and treatment services in the public and private sector in Kenya and evaluate patients’ ability to pay for services along the continuum of care. We focused on the high-burden NCDs and their risk factors, and we included cardiovascular diseases, diabetes, breast and cervical cancer, and respiratory diseases in the assessment. These are the high prevalence diseases that the World Health Organization is targeting to achieve a 25% relative reduction in the overall NCD mortality by 2025 [1].

## Methodology

We performed a comprehensive assessment of patient payments related to the four targeted NCDs—cardiovascular disease, cancer, diabetes, and respiratory disease—we created a matrix of relevant clinical services along the continuum of care of each disease or key risk factor. We focused on risk factors and disease groupings for which there were comprehensive guidelines for early detection and management. An additional key consideration was inclusion of the risk factors and disease groups targeted in Kenya’s NCD strategic planning documents, including the Kenya Health Policy 2014–2030 [14], the Kenya National Strategy for the Prevention and Control of Non-Communicable Diseases 2015–2020 [15] and the National Cancer Control Strategy 2011–2016 [8]. Table 1 provides a summary of the health services included in this study.

**Table 1:** Targeted diseases, risk factors, and medical services.

	Screening	Diagnosis	Medication Management	Treatments and Complications Included in Estimates
Hypertension	**X**	**X**	**X**	Complications including stroke, acute myocardial infarction, angina, heart failure, chronic kidney disease, diabetic foot, and retinopathy
Diabetes	**X**	**X**	**X**	
Cervical and Breast Cancer	**X**	**X**	**X**	Cancer treatment by stage at diagnosis
Asthma and COPD		**X**	**X**	Hospital admissions for management of symptoms (acute episodes)

X indicates that the service was included in the cost estimation

### Developing frameworks to identify services provided

We developed individual frameworks or treatment pathways for each health service identified in Table 1. Each framework included, as appropriate, physician visits, diagnostic procedures, laboratory tests, medications, and hospitalization. As a first step, the clinical pathways required for each framework were developed through a detailed review of the published Kenya guidelines. These included the Clinical Management and Referral Guidelines (Volume III): Clinical Guidelines for Management and Referral of Common Conditions at Levels 4–6 Hospitals (2009) [16]; the Guidelines for Asthma Management in Kenya (2011) [17]; the National Guidelines for Prevention and Management of Cervical, Breast and Prostate Cancers (2012) [18]; the National Guidelines for Cancer Management Kenya (2013) [19]; the National Palliative Care Guidelines (2013) [20]; and the Non-Communicable Diseases Clinical Guidelines for Clinical Officers and Nurse Task-Shifters (Médecins Sans Frontières [MSF] Belgium and Kenya Mission Kibera Project 2015, version 3.0) [21]. Second, to ensure that the guidelines reflected real-world practice, the guideline-directed frameworks were presented to a wide range of stakeholders, including clinicians from various specialties, such as doctors, nurses, and pharmacists. These interactions with providers were also used to collect details on the number of units of service typically required and estimates on the distribution of patients by disease severity. We interviewed three cancer treatment specialists, two cancer screening specialists, one surgical nurse, one intensive care unit nurse, two cardiologists, three diabetes management specialists, three palliative care specialists, one general NCD specialist, one asthma and COPD specialist, and two ophthalmologists. As a third step, we reviewed published literature [22–25] to confirm the itemization of services in the frameworks and the distribution of patients by severity or stage of disease (when clinical pathways differed by severity). In the fourth and final step, we obtained data from literature, population-base sources (for example, cancer registry) and clinic databases where available, on severity and stage of disease. For breast and cervical cancer, we used data on cancer stage at diagnosis from the Nairobi Cancer Registry. For hypertension, diabetes and asthma, we utilized data from the provider sites and published literature [22–23] to represent the public sector and private practitioner self-reported distribution in their practice for the private sector. For inpatient procedures including cardiovascular treatments, we used information available in the published literature supplemented with expert opinion [24,25]. The data from steps 2, 3 and 4 were used to weight the individual costs by severity or stage of disease in order to estimate the weighted average cost for the disease when applicable. This was performed to estimate average cost of a disease, when applicable, as the cost of treatment differs by severity of the condition.

### Estimating patient payments in the public and private sectors

We generally employed an itemization cost approach using standard economics methods [26–27] to quantify patient payments for each clinical procedure or service using the frameworks. The perspective employed in the cost estimation is that of the patient, as the objective is to estimate patient payments for NCD services in the public and private sectors. For example, for management of diabetes, the framework would include physician visits, cost of medications and insulin when required, and any diagnostic procedures required for patient monitoring. The number of each type of service required in any typical year was determined through consultation with clinicians to estimate the cost for an annual period.

To reflect the public-sector payments, we collected data from Kenyatta National Hospital, the main tertiary referral hospital (level 6), located in Nairobi, with a capacity of 1,800 beds. Patient payments were obtained from the finance department for each service category, including clinic visit cost, diagnostic tests, and procedures. The hospital’s main pharmacy provided costs for the medicines. Patients pay the full acquisition cost of medications, which are based on individual medication distributor prices with a mark-up of 10% on all medication except cancer medicines with 0% mark-up, and a dispensing cost of U.S. $0.20 per prescription.

In addition to the standard public sector payments, we also collected costs that reflected care provided in a quasi-public facility such as facilities run by faith-based organizations and international agencies. We selected MSF Belgium’s Kibera South Health Center (MSF-KSHC), which served an informal settlement area in Nairobi, as an example of a quasi-public facility. These institutions do not receive state funds but are private facilities that behave like public facilities. The MSF facility, with approximately 500 outpatient visits daily, provided services and medications free of charge. Other quasi-public facilities in Kenya though may charge nominal payments for services provided. To reflect the payment that patients are likely to pay at quasi-public, not-for-profit private clinics, we estimated the cost to provide care to patients at the MSF-KSHC assuming payments would cover cost of operations to ensure sustainability of services. We used an ingredient-based approach and estimated the cost of staff time, laboratory procedures, and medications using the frameworks described above as guidance for each targeted medical service. Staff time was estimated by interviewing clinicians and direct observation of the staff engaged in patient interaction. Staff cost was calculated by multiplying time expended by the hourly wage for each individual staff member. The unit costs for reagents (ingredients) for laboratory procedures and medications were provided by the laboratory and pharmacy managers respectively. At MSF-KSHC, we estimated the cost of hypertension, diabetes, and asthma management along with cervical cancer screening. Patients with complications were referred to Kenyatta National Hospital, and therefore, the same payments as those paid by patients receiving services at Kenyatta National Hospital apply to other types of medical procedures.

For private sector payments, patient costs for visits, laboratory tests, diagnostic procedures, and inpatient stays (basic ward bed) were obtained from private-sector practitioners (generally two clinicians for each disease area) and hospitals at the same level as Kenyatta National Hospital in terms of service provision (information was collected from two hospitals in Nairobi). We used prices published in the Drug Index [28], which provides distributor prices for medication brands available in the Kenyan market, to estimate the cost of medications in the private sector. We reviewed pharmacy retail prices (from stand-alone outlets and hospitals) for selected medications and found them to be highly variable—both higher and lower than the published Drug Index price list. Based on consultation with our medical experts, we used the cost of commonly prescribed brands listed in the Drug Index. These prices were reviewed and confirmed by the study team of clinicians, economists and health sector managers.

Microsoft Excel 2013 was used to develop the frameworks and compute the payment estimates. The rows comprised various diagnostic examinations, laboratory tests, and treatments for diseases, and the columns included other costing variables, such as estimated percentage of patients receiving the intervention, the type of personnel providing the service, time taken to provide the service, formulation and size of the product, frequency of dose per day and number of days the treatment was given, number of units necessary, cost per unit, and derived total cost of the health service. Payment data were entered into the frameworks as applicable with the aim of arriving at total costs of each diagnostic examination, test, or treatment. The unit costs were multiplied by the number of units necessary for the full course of management or treatment which generally included an annual period for outpatient care and complete inpatient episode for hospital admissions. When there were differences in treatment by severity of disease or type of patients, except opinion and data available from literature were used and an estimated proportion of patients receiving each particular treatment algorithm was identified to compute a weighted average total cost of management for the typical individual with the condition. Using the same process, payments were estimated for NCD care in the public and private sector. For the public sector, when applicable, we report the average (mean) cost for outpatient patient management, combining estimates from Kenyatta National Hospital and the MSF-KSHC facility. Most of the payment estimates were very similar; in a few cases, there were large differences, which were mainly due to the variation in the type of diagnostic tests, procedures, or medications used for patient management and their differential cost. Most of the costs in the private sector were similar, expect for medications as indicated previously. All costs were calculated in 2017 Kenyan Shillings and converted to U.S. dollar values (at 100 Kenya Shillings per U.S. dollar) for reporting. Cost data was collected from March 2016 and October 2016. We only report patient payments in this study.

### Generating affordability metrics

To assess affordability, we report key metrics on household expenditure, annual salary in the formal sector, insurance coverage, and annual out-of-pocket payments for health care. These estimates were derived from secondary data analysis of recent Kenyan national surveys [13,29–32] and include data from the 2014 Kenya Demographic and Health Survey and the 2016 Kenya National Bureau of Statistics (KNBS) Economic Survey. We used previously developed analytic variables (no new variables were created for this study from these secondary data sources; see references for details on methodology) and present estimates stratified by key characteristics of interest. For example, we present insurance status by type of employment based on whether person worked in the formal or informal sector. To fully explore potential disparities by socioeconomic status, we report insurance status and catastrophic spending by wealth quintiles (a quintile is 20% of the population). We used information from the 2013 Kenya Household Health Expenditure and Utilization Survey [31], and catastrophic spending was defined as out-of-pocket spending greater than 40% of household non-food expenditure.

This study did not involve human subjects’ research. No patient level data was analyzed and no identifiable information was collected. We obtained payment information from public and private facilities and practitioners, analyzed data from reports published by the government of Kenya and performed a review of the relevant literature. This study was approved by the Kenyan Ministry of Health and Kenyatta National Hospital.

## Results

### Screening and diagnosis of NCDs in Kenya

Table 2 presents estimated patient payments for screening procedures for early detection of NCDs. These costs range from $3.90 to $10.50 in public facilities and $18.00 to $36.00 in private facilities. Public-sector costs could be as low as $2.01, which is the cost of a health care visit for many screening procedures. Screening can also be performed in combination with other visits at potentially no additional cost to the patient; that is multiple services can be provided for a single visit fee. Pap smear for cervical cancer screening and random blood sugar for diabetes screening cost more than the basic consultation payment, as they include laboratory testing as well.

**Table 2:** Patient costs for screening and diagnosis of NCDs (2017 US dollars).

	Public Facilities^a^ (U.S. $)	Private Facility (U.S. $)
**Screening**		
Breast cancer (clinical breast exam-CBE)	3.90	18.00
Cervical cancer—Visual Inspection with Acetic Acid/Lugols Iodine (VIA/VILI)	3.90	–^b^
Cervical cancer—pap smear	10.50	25.00
Hypertension—2 or 3 blood pressure readings	8.52	36.00
Diabetes—random blood sugar	4.95	19.00
**Diagnosis (when further evaluation after screening is required or when patients present with symptoms)**		
Breast cancer	401.00	1,205.24
Cervical cancer	181.38	548.39
Hypertension	31.81	127.96
Diabetes	41.95^c^	382.91
Asthma	4.23	53.00
Chronic Obstructive Pulmonary Disease (COPD)	17.50	110.00

^a^ Estimates are average (mean) cost at public hospital and low-cost quasi-public health clinic. Screenings are done during a single visit.

^b^ VIA is generally not offered in the private facilities in Nairobi, Kenya; VIA is provided only in government and quasi-governmental facilities (for example, MSF and faith-based clinics)

^c^ There is a wide range in costs of diabetes diagnosis due to the varied options and combinations of laboratory tests routinely done at different facilities.

Diagnostic procedures are generally more expensive than screenings, while the cost of diagnosing cancers is substantially higher than screening for cancer. Cervical and breast cancer diagnostic procedures are estimated as $181.38 and $401.00 in the public sector and $548.39 and $1,205.24 in the private sector. Patient payments for diagnosing diabetes and treatment planning can also be a substantial outlay for the average Kenyan, with an estimated cost of $41.95 in public facilities and $382.91 in private facilities.

### Management of hypertension, diabetes, asthma, and COPD

Table 3 shows the estimated costs to patients with hypertension, diabetes, asthma, and COPD. For hypertension, we categorized patients by the number of antihypertensive drugs prescribed and whether resistant hypertension was present despite receiving standard medication management. The distribution of patients is provided in this table, and the majority of the patients were on two-drug therapy. We did identify differences in the distribution of patients by number of hypertension medications; for example, the proportion on two-drug therapy was 35% and 70% respectively in public and private facilities. Additionally, asthma patients were categorized as mild or severe, and the pattern of distribution between these groupings was similar in the public and private sector. Patient costs for care in public facilities ranged from $25.64 to $372.45, and the range for private facilities was $295.45 to $1,530.06.

**Table 3:** Patient costs for hypertension, diabetes, asthma and COPD management (2017 US dollars).

	Public Facility^a^		Private Facility^a^	
	Percentage of Patients	U.S. $	Percentage of Patients	U.S. $
Hypertension				
Treatment—1 drug	20	25.64	5	418.20
Treatment—2 drug	35	67.25	70	596.44
Treatment—3 drug	25	81.20	15	948.06
Treatment—4 drug	10	110.33	–	–
Treatment—resistant^b^	10	159.36	10	987.17
Diabetes^c^				
Insulin only	32	186.40	10	541.22
Oral medication only	25	88.61	65	488.60
Both insulin and oral medication	43	234.44	25	675.85
Asthma				
Mild	95	67.93^d^	95	295.45
Severe	5	146.74^d^	5	879.08
Chronic Obstructive Pulmonary Disease (COPD)	100	372.45	100	1,530.06

^a^ The costs in this table include physician consultations for the average patient, medications, and admissions for managing symptoms (hypoglycaemia or status asthmaticus). Major complications such as stroke are not included in this table;.

^b^ Patient has high blood pressure despite the use of combination medications.

^c^ The proportions reported in the public facilities are based on a recent study [21] while the proportions in the private facilities are based on expert opinion.

^d^ There is a wide range in asthma management costs which reflects the variation in products, formulations, and brand-name medications used routinely at different facilities. Approximately 5% of asthmatics have severe asthma and require specialized treatment administered at health facilities and even admissions.

### Cervical and breast cancer treatment

Table 4 presents patient costs for breast and cervical cancer treatment by stage at disease. The cost includes all guideline-suggested treatments, including surgery, radiation, chemotherapy, and hormonal therapy (for hormone receptor–positive breast cancer). Treatment for breast cancer stages I and II followed similar clinical recommendations and therefore had the same estimated cost. The costs of treating stage III breast and cervical cancer depended on whether the treatment followed a curative approach or a palliative approach. The cost of treating cancer generally increased by stage; the exception was some stage III and most stage IV cancers. Some patients at this stage only receive non-curative palliative care and therefore incur a lower cost than patients at stages with curative treatment. Public-sector patient cost for treating stage I, II, and III breast cancer ranged from $1,340.38 to $1,542.58, and the cost for cervical cancer ranged from $841.50 to $1,575.93. Breast and cervical cancer treatment in the private sector was generally almost 10 times more expensive than in the public sector. Palliative care for a 6-month period was $169.20 and $752.43 in the public and private facilities respectively.

**Table 4:** Patient costs of cervical and breast cancer treatment (2017 US dollars).

	Percentage of Patients^a^	Public Facility (U.S. $)	Private Facility (U.S. $)
**Breast Cancer Treatment**^**b**^			
Stage I	7	1,340.38	10,914.45
Stage II	35	1,340.38	10,914.45
Stage III (curative approach)	19	1,542.58	11,862.36
Stage III (palliative approach) and Stage IV	40	675.35	8,569.87
**Cervical Cancer Treatment**			
Stage 0 (carcinoma in situ)	1	85.50	257.25
Stage I	16	841.50	7,369.90
Stage II	36	962.50	7,669.90
Stage III (curative approach)	17	1,575.93	7,866.90
Stage III (palliative approach) and Stage IV	30	349.20	3,734.39
**Palliative Care**^**c**^		169.20	752.43

^a^ Distribution of patients by stage was obtained from the Nairobi Cancer Registry.

^b^ Hormonal therapy would follow the initial breast cancer treatment, depending on the tumor profile and patient characteristics. Tamoxifen cost was U.S. $0.10 per day.

^c^ Palliative care includes pain and symptom management as well as psychosocial support to the patients and their families. Patient payments for an average duration of 6 months is presented.

### Managing common complications of hypertension and diabetes

Uncontrolled hypertension and diabetes can lead to serious complications that are very expensive to manage. Table 5 summarizes the cost of some of the most common complications requiring hospitalization, including strokes, acute myocardial infarction (heart attack), angina, and heart failure. The cost to patients ranged from $1,026.07 to $1,995.65 and $2,160.51 to $16,710.82 in the public and private facilities, respectively. Chronic kidney disease requiring dialysis would require annual payments of $5,338.00 in the public sector and $11,024.00 in the private sector. Similarly, the cost for kidney transplant was $9,237.00 in the public center versus $19,724.00 in the private center. Diabetic foot care and retinopathy requiring outpatient management were less than $100 per episode in the public sector and more than double that cost in the private sector.

**Table 5:** Patient costs of managing complications of hypertension and diabetes (2017 US dollars).

	Cost per Inpatient or Outpatient Episode	
	Public Facility (U.S. $)	Private Facility (U.S. $)
Stroke	1,873.93	16,710.82
Acute myocardial infarction	1,995.65	12,529.47
Angina	1,236.81	10,740.44
Heart failure (secondary to hypertension)	1,026.07	2,160.51
Chronic kidney disease (dialysis)^a^—70% of patients	5,338.00	11,024.00
Chronic kidney disease (transplant)—30% of patients	9,237.00	19,724.00
Diabetic foot^b^	69.95	731.99
Diabetic retinopathy^b^	94.45	242.68

^a^ Dialysis costs includes an average of two sessions per week per patient for 1 year.

^b^ Diabetic foot and diabetic retinopathy payments include one unilateral episode.

### Affordability of screening, diagnosis, and treatment of NCDs in Kenya

Table 6 summarizes key health care affordability metrics, including annual household expenditure, average earnings in the formal sector, and insurance coverage. The estimated average household expenditure per adult in 2013 was $412.80, $721.20 in the urban areas and $272.40 in rural settings [29]. The average earning per employee in the formal sector, which accounts for about 17% of the total employment, was approximately $6,000 annually in 2016 [30]. Most Kenyans aged 15 to 49 years do not have health insurance (82% of women, 79% of men). There is almost universal health insurance coverage among those employed in the formal sector, whereas less than one-fifth of those working in the informal sector have coverage [33]. Among those with health insurance, most are covered by the NHIF [13], and a very small proportion receives employer-based insurance [33].

**Table 6:** Health care affordability metrics in Kenya (2017 US dollars).

Measure	Estimate	Year of Data	Data Source
**Annual Household Expenditure per Adult (U.S. $)**			
Average	412.80	2013 (year published)	KNBS and Society for International Development [29]
Urban (31.2% of the population)	721.20		
Rural (68.8% of the population)	272.40		
**Average Annual Earning per Employee in the Formal Sector (U.S. $)**			
Private	5,952.12	2015	KNBS Economic Survey [30]
Public	6,264.09		
**Health Insurance Coverage (%)**			
Overall (15–49 years)	18% of women, 21% of men	2014	Kenya Demographic & Health Survey, [13]
NHIF	14% of women, 18% of men		
Employer-based coverage	2% of women, 3% of men		
NHIF—Formal sector (17% of workforce)	98% coverage for this workforce	2014	U.S. Agency for International Development (USAID) and Health Finance & Governance, [33]
NHIF—Informal sector (83% of workforce)	16% coverage for this workforce		

**Fig 1:**
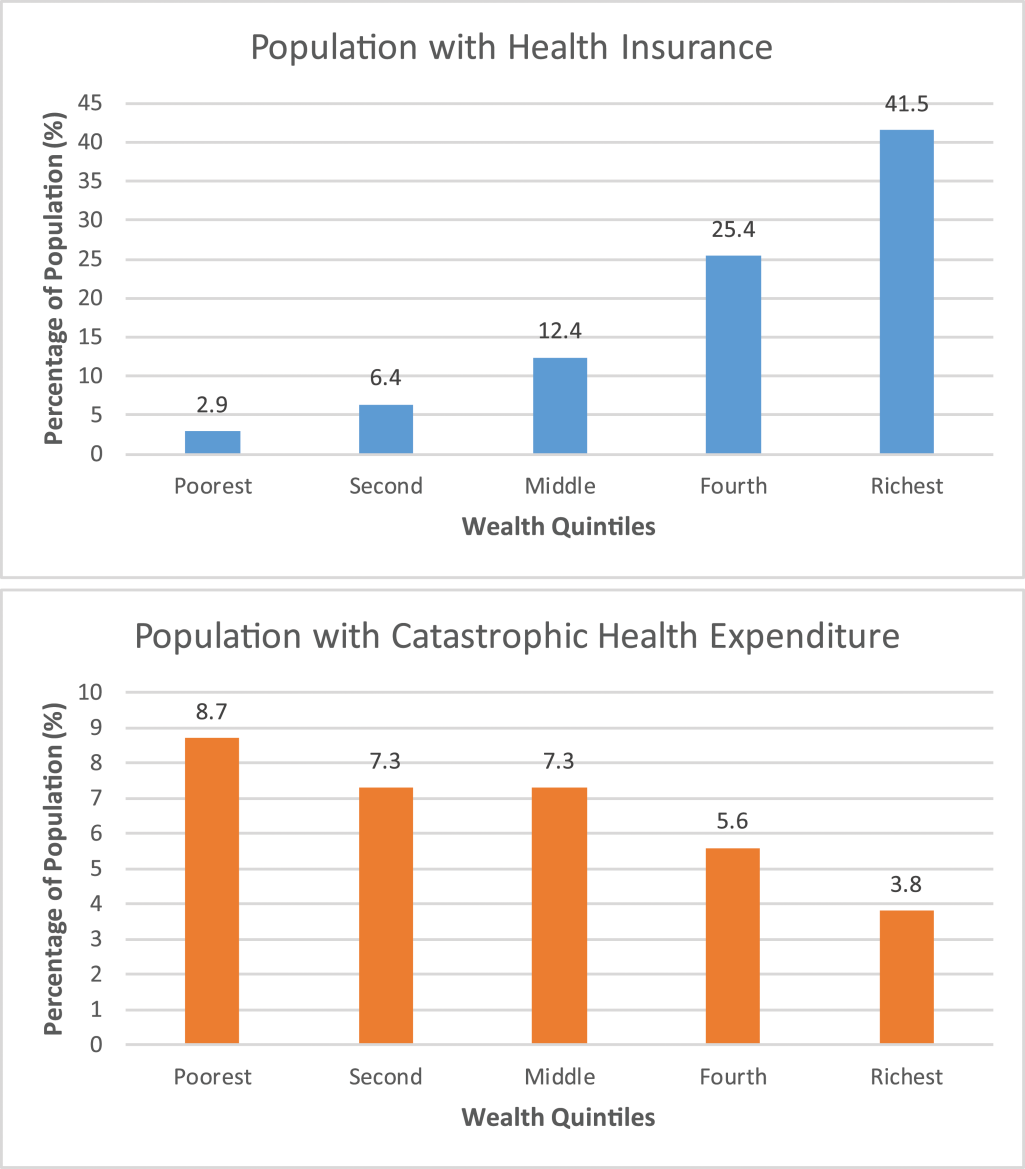
Catastrophic health expenditure: Out-of-pocket spending ≥ 40% of household non-food expenditure. Source: 2013 Kenya Household Health Expenditure and Utilization Survey [31].

Fig 1 shows the proportion with health insurance and catastrophic spending by wealth quintiles. There is a consistent inverse relationship between health insurance coverage and catastrophic health expenditure. Those in the poorest quintile had the lowest proportion with health insurance (2.9%), and those in the wealthiest quintile had the highest proportion (41.5%). Conversely, the poorest individuals had the largest proportion of catastrophic spending (8.7%), and the wealthiest individuals had the lowest proportion (3.8%) [31]. It is interesting to note that in each of the wealth quintiles, at least some households experienced catastrophic health expenditure; the poor are disproportionally impacted, but the wealthy can be vulnerable to financial collapse as a result of high health expenditures as well. On average, medication management for hypertension (2 drug combination) or diabetes would require about 1%-2% and 8%-10% of the average annual income (reported in Table 6) respectively in the public and private sectors. For curative breast cancer treatment, approximately one-fifth of the annual income will be required for care in public facilities and more than 1.5 times the annual income for care in private facilities.

## Discussion

Cost and affordability are key emerging issues in addressing the burden posed by NCDs in LMICs. In this paper, we report on patient payments required for obtaining health care services in Kenya along the continuum of care from screening, to diagnosis, to treatment in public- and private-sector facilities. Screening costs were generally inexpensive, with many tests available for about $4. The costs of diagnostic procedures for follow-up of abnormal screening results, though, were substantially higher. For example, payments for breast cancer diagnosis were on average $401 in public facilities and $1,205 in private facilities. The payments required to undergo many diagnostic tests, specifically those related to cancer and diabetes, are beyond the reach of the average Kenyan, as the annual household expenditure per adult is about $413. Overall, the payments for screening and diagnosis were consistently lower in public-sector than private-sector facilities, but even these subsidized costs are likely to pose access barriers to the majority of Kenyans.

Management of hypertension, diabetes, and asthma required modest payments in the public sector, ranging from $26 to $234. These payments are far more affordable than the charges in private facilities, which ranged from $418 to $987. Inpatient treatments required substantially higher payments. Patient payment for breast and invasive cervical cancer treatment in the public sector was about $1,000 but more than $7,000 in the private sector. Stroke admissions ($1,874 versus $16,711) and annual cost of dialysis for chronic kidney disease ($5,338 versus $11,024) were among the most expensive treatments. Kidney transplants would have a very high initial cash outlay but potentially more cost effective in the long run.

There is very limited information on cost of NCD treatments in Sub-Saharan Africa that can be used to compare the findings from this study [34–37]. One study from South Africa reported stroke management cost of $16,993 [34] which is very similar to the cost of $16,711 reported in the private sector in this study. Another study from Sudan estimated kidney transplantation to cost $18,132 while our estimates range from about $9,000 in the public facilities to $20,000 in the private facilities [35]. An evaluation of diabetes foot ulcer treatment in Nigeria [36] reported a total cost of $1,619 for the entire course of management while in this study we report costs of about $70 and $732 for public and private facilities respectively for a single episode of care. These estimates are not directly comparable as the reason for the higher cost in the Nigerian study is because their estimates include multiple episodes and the cost of foot amputation (for a proportion of patients requiring this), which is not included in this study. Overall, the findings from our study mirror past cost estimates from Sub-Saharan Africa but differences between the types of procedures and follow up periods included in the studies make systematic comparisons of cost estimates difficult to perform.

The health insurance information presented in this study show that most Kenyans are not covered [32]; therefore, they have to use savings or borrow funds to pay for health care. In 2014, approximately 20% had health insurance coverage through the NHIF; there are ongoing efforts to increase NHIF enrollment but nevertheless a large proportion of the population remains uninsured. [38] The fund provides coverage for several types of high-cost diagnostic tests and treatments, including $80 for a CT scan, $150 for an MRI, $250 per chemotherapy session, $36 per radiotherapy session, and up to $1,300 for surgeries. In general, NHIF payments will cover inpatient admissions for NCDs and specific diagnostic tests at capped rates. NHIF currently do not cover most medication costs, many diagnostic tests, costs of managing complications, and palliative care (for example, costs of chemotherapy ports and stoma bags).

The low rate of health insurance coverage and high cost of many NCD treatments may result in health care disparities in Kenya. Poor households are far more likely not to have health insurance and are also more likely to experience catastrophic health expenditure compared with wealthy households. The statistics reported in this manuscript on catastrophic expenditure reflect all health care costs. The gap between the rich and poor is likely to increase, as the burden from NCDs is projected to grow across all socioeconomic groups because of the high prevalence of risk factors [7]. Additionally, NCDs are expected to result in overall higher household financial burden; a recent study reported that although general illnesses reduce household income by 13.63%, NCDs reduce household income by 28.64% [39]. An increase in health insurance coverage among those in the informal sector can help offset some of this anticipated financial burden. Workers in the informal sector have been encouraged to make voluntary contributions to NHIF in monthly installments of $5 or annual contributions of $60 per family. The current low levels of voluntary contributions need to be systematically explored, and research is needed to better understand barriers to obtaining health insurance coverage.

Currently, screening for cervical and prostate cancers is a covered service by NHIF, but the public awareness of this benefit is unknown as there are no specific studies on this issue. Evaluating the current level of knowledge and adopting policies, such as systematic patient and provider education, to increase awareness of the inclusion of screening within the basic benefit package would be essential to reduce the burden from NCDs. Most NCDs are not diagnosed at an early stage, and screening should be implemented for other NCDs, including hypertension, diabetes and breast cancer, to identify risk conditions and diseases early, when treatments are more effective. Early detection of NCDs and prompt management is key to mitigate the high cost of advanced disease and complications. To encourage compliance with follow-up recommendations, any coverage provided for screening should also include follow-on diagnostic tests and medication management. A recent national survey reported that only 22% of those diagnosed with high blood pressure were taking the medications prescribed by a health worker [7]. Reducing financial barriers may encourage more individuals to comply with provider recommendations. In addition to screening and diagnosis, prevention of NCDs should be prioritized. The World Health Organization has identified several highly cost-effective initiatives, including interventions to increase physical activity, promote healthier diets, reduce tobacco use, and eliminate harmful use of alcohol [1]. These lifestyle changes should be vigorously promoted to reduce incidence of NCDs and research should be undertaken to identify the best approaches to implement these health promotion behaviors. Prevention is important as it can potentially reduce the burden from NCDs and the related cost of financing high-cost NCD inpatient treatments.

In Kenya, NCD services are provided in both the public and private sectors, but the payments required for services differ substantially. The public sector is subsidized through government payments, and therefore, it is expected to provide care at lower cost to patients. However, no systematic comparison of the quality of the services provided in private and public facilities is available to assess the value of the care provided. Past research exploring quality of care and access in the public and private health sectors in LMICs has highlighted the complexity of such comparisons due to methodological challenges and wide variation across providers [40–43]. In Kenya, this is certainly also the case, as the public sector requires lower patient payments but, in some instances, does not provide all the required NCD services or procedures (for example, hormone receptor status testing and percutaneous coronary angioplasty). There also may be long waiting lists for NCD treatments. The private sector requires higher payments but may offer more comprehensive and timely management of NCDs. We need to better understand the role of public- and private-sector facilities in delivering NCD health services in Kenya, and this will require better data collection to assess the quality of the care provided.

The payments reported in this study represent the average cost to patients to obtain NCD health care services in the public- and private-sector facilities. A potential limitation of this study is that patient payments, especially in the private sector, can vary based on the type of provider, location, and other factors. There may also be variability in the payments required at different levels of public facilities, for example tertiary versus district level. We report the estimated patient payments as we did not collect actual payments made by individual patients. Furthermore, we report payments made by patients to evaluate affordability and not the “true cost” of providing the services as payments include varying levels of mark-ups in the private sector and subsidies in the public sector. Cost of medications in the private sector is based on information available in the Drug Index and large variation in medication prices exist across pharmacies. Drug Index are wholesale or distributor prices and therefore some patients may be paying more than the estimated price while others may be paying less, due to variations in mark-ups and medicine procurement practices across pharmacies. In addition, the payments reported for inpatient admissions represents the initial episode of care and does not systematically account for all post-discharge complications, long-term disabilities, and comorbidities related to the treatments. Additionally, non-medical costs borne by patients to receive required health care services are not included. These costs could be substantial for patients who have to travel to tertiary care centers to receive specialized treatment for NCDs. Furthermore, the cost estimates do not include loss of income due to disabilities and the time required for treatments. Therefore, the true cost to patients who undergo NCD treatments is likely to be much higher than the health care payments presented in this study.

## Conclusion

In Kenya, the high cost of NCD treatment and low rate of health insurance coverage significantly limits affordability for most of the population. In the short term, efforts should be made to increase insurance coverage among those employed in the informal sector, but at the same time, prevention and screening interventions should be prioritized to reduce the financial burden to the nation as a whole. NCD health care services are provided in both the private- and public-sector facilities in Kenya, and the value of these services, benefit gained for the cost expended, should be systematically assessed in future studies.
